# Logics of acquiring medicines from informal retailers in four African countries

**DOI:** 10.1080/16549716.2025.2574764

**Published:** 2025-10-29

**Authors:** Janelle M. Wagnild, Samuel Asiedu Owusu, Simon Mariwah, Victor I. Kolo, Ahmed Vandi, Didacus Bambaiha Namanya, Rutendo Kuwana, Babatunde Jayeola, Kate Hampshire

**Affiliations:** a Department of Anthropology, Durham University, Durham, UK; b Directorate of Research, Innovation and Consultancy, University of Cape Coast, Cape Coast, Ghana; c Faculty of Social Sciences, Department of Geography and Regional Planning, University of Cape Coast, Cape Coast, Ghana; d School of Public Health, University of Medical Sciences, Ondo City, Nigeria; e Department of Community Health and Clinical Sciences, Njala University, Bo, Sierra Leone; f Environmental Health Department, Ministry of Health, Kampala, Uganda; g Market Surveillance and Control Team, World Health Organization, Geneva, Switzerland; h Medicines, Diagnostics, and Infrastructure, World Health Organization Regional Office for Africa, Brazzaville, Republic of Congo

**Keywords:** Ghana, Nigeria, Sierra Leone, Uganda, medicine quality

## Abstract

**Background:**

In sub-Saharan Africa and other low-income contexts, informal medicine markets are widespread. Understanding the drivers of consumer demand is important, especially given the concerns and risks associated with medicines in the informal sector.

**Objectives:**

This study aims to 1) describe the informal medicine sector in four anglophone African countries, and 2) understand why people patronize informal medicine sellers.

**Methods:**

Participant observation was conducted in eight markets (37 market stalls) across Ghana, Nigeria, Sierra Leone, and Uganda, supplemented by data collected during focus group discussions (with *n* = 611 participants) and key informant interviews (with *n* = 111), in which we discussed where participants got medicines in their communities and underlying reasons.

**Results:**

We identified four distinct groups of actors in the informal medicine sector: sellers at weekly markets, itinerant peddlers, roadside sellers, and operators of general provision shops. There were multiple rationales for patronage of informal sellers that varied depending on the context, including flexibility in payment options, convenience and accessibility, and social/cultural drivers. Importantly, there were tradeoffs and tensions between these drivers that participants had to negotiate within the contexts of their current circumstances.

**Conclusions:**

These findings suggest that the informal medicine market is segmented and complex, and that patronage is driven by multiple logics that are rooted in gaps in formal healthcare provision. Regulatory measures therefore need to go hand-in-hand with efforts to address these gaps and expand effective access to quality-assured medicines through [inter alia] offering more flexible modes of payment, reducing public-sector medicine stock-outs, and improving patient-physician trust and communication.

## Paper Context

- **Main findings:** The informal medicine market in anglophone African countries (Ghana, Nigeria, Sierra Leone, and Uganda) is complex, and patrons have multiple (and often competing) reasons for purchasing medicines from these outlets.
- **Added knowledge:** Most reasons for patronage stem from difficulties faced with securing quality-assured medicines from ‘official’ sources (e.g. unaffordability and inaccessibility), particularly for the poorest and most rural segments of the population.
- **Global health impact for policy and action:** Addressing limitations to medicine access within the formal sector using an equity-informed approach will reduce the demand for medicines in the informal sector.

## Background

In many lower- and middle-income countries (LMICs), and sub-Saharan Africa in particular, the informal sector plays a key role in supplementing the formal health care system [1–4]. Accessing medicines via the public sector can be challenging, as government facilities often struggle to meet demand, in terms both of personnel and sufficient medicine supplies for the populations they serve [5,6]. Frequent public-sector medicine stock-outs drive many people to the private sector: an eclectic range of medicine providers, from licensed pharmacies and over-the-counter retail outlets, to various kinds of more informal operators. Often, the boundaries between formal and informal providers are not always clear [7], as retailers operate with varying degrees of regulation and legality [8].

Following the World Health Organization’s definition [9], we define the ‘informal market of medical products’ as a sector where: 1) The manufacture, import or export, distribution, sale, supply, or purchase of medical products takes place outside of the legal, regulatory, or administrative oversight of relevant public health or regulatory authorities; 2) The medical products have or have not been assessed for safety, efficacy, or quality by public health and regulatory authorities; and 3) the aforementioned activities may be conducted by persons or entities with or without appropriate qualifications, and may take place in a physical, virtual, or a hybrid environment.

Informal vendors are thus those who sell medicines ‘outside the legislative and administrative framework imposed by a country’s government and biomedical health system’ [10]. This includes, for example, itinerant drug peddlers and those selling pharmaceuticals within open markets or general provision shops.

There is a large presence of informal medicine sellers throughout sub-Saharan Africa, which poses serious concerns about medicine quality and antimicrobial resistance. Several studies have shown that medicines sold in informal/unlicensed outlets are more likely to fail quality standards than those by licensed retailers [11,12]. Additionally, obtaining medicines from informal sellers has been linked to inappropriate use of antimicrobial medicines in particular [13], through dispensing without prescriptions, supplying incorrect/inappropriate products, or selling incomplete doses. Poor-quality medicines and improper dispensing practices may cause direct public health harms and can significantly contribute to antibiotic and antimalarial resistance [14–17]. Thus, the proliferation of the informal medicine sector is a serious public health concern.

Although part of the growth of the informal medicine market is attributable to weaknesses in governance and regulation in these resource-constrained contexts, their prevalence and success indicate they are, at least in some ways, effectively meeting consumer demand. Little work, particularly using qualitative methods, has sought to describe informal medicine sellers and understand the reasons people have for patronizing them [8]. Existing work in sub-Saharan Africa, predominantly in francophone countries, has indicated that relative affordability, convenience, and social/cultural factors are key reasons people buy medicines from informal medicine sellers [4,10,12,18,19]. Establishing these broad parameters of patronage is an important starting place, but it is important to recognize that the informal sector is very diverse, with multiple types of sellers (e.g. itinerant peddlers, regular market-stall holders, etc.), scale of operation, and social/geographical contexts [18]. The drivers of demand may thus be equally diverse; understanding these has been identified as a key research and public health priority [8,20].

To this end, this study aims to 1) describe the informal medicine sector in four anglophone African countries, and 2) understand why people patronize informal medicine sellers, with careful attention paid to variation in contexts and rationales.

### Medicine systems in the four countries

Details about the medicine systems in each of the four countries are provided elsewhere [21]. Briefly, public-sector healthcare in Ghana, Nigeria and Sierra Leone incurs user fees for consultations plus the costs of any prescribed medicines, except for those covered by insurance in Ghana and Nigeria or those with an exemption in Nigeria and Sierra Leone (e.g. children under the age of five); accessing public-sector services is free of charge in Uganda. In the private sector, medicines are legally available in licensed pharmacies and licensed over-the-counter medicine retailers.

## Methods

### Study context and design

The data reported here were collected as part of a broader study on perceptions and practices around substandard and falsified medical products in Ghana, Nigeria, Sierra Leone, and Uganda [21]. Four study sites per country were purposively selected: one urban and one rural in each of the two geographically- and culturally distinctive regions (Table 1). Qualitative fieldwork, combining ethnographic observation, in-depth interviews, and focus group discussions, was conducted in each study location between November 2022 and October 2023. Data were collected by an experienced research team representing the four African countries, the UK, and the USA.

**Table 1.** Summary of research activities across the study sites.

	Participant observation activities	FGD participants (n)	Key informants (n)
**Ghana**		**137**	**21**
*Greater Accra*			
Urban		29	5
Rural		27	5
*Upper East*			
Urban	Market observations (*n* = 3)	36	8
Rural		45	3
**Nigeria**		**174**	**35**
*Federal Capital Territory*			
Urban	Market observation (*n* = 1) Roadside vendor observation (*n* = 1)	48	10
Rural		64	8
*Kwara State*			
Urban		48	9
Rural		14	8
**Sierra Leone**		**115**	**39**
*Western Area Rural District*			
Urban		27	3
Rural		21	4
*Port Loko District*			
Urban		16	17
Rural		33	2
*Bo District*			
Urban	Market observation (*n* = 1)	NA	2
Rural		18	10
*Kenema District*			
Urban	Market observation (*n* = 1)	NA	1
**Uganda**		**185**	**16**
*Mbale*			
Urban		51	4
Rural		53	4
*Mbarara*			
Urban	Market observation (*n* = 1)	39	4
Rural		42	4
*Kampala*			
Urban	Market observation (*n* = 1)	NA	NA

### Data collection methods

This paper focuses particularly on data from ethnographic observations undertaken at informal markets in each of the countries. In each study site, surrounding markets were sampled based on the study participants’ indications that medicines might be sold there. Eight informal open markets in urban areas were thus purposively sampled and visited, in which we observed a total of 37 stalls selling medicines: 3 markets (17 stalls) in Ghana, 2 markets (17 stalls) in Sierra Leone, 1 market (3 stalls) in Nigeria and 2 markets (no stalls) in Uganda. Additionally, one grouping of urban roadside vendors in Nigeria was visited. At each market/stall, research team members approached the stall-holder to explain the research objectives and seek informed consent for participation. The researchers then observed what pharmaceuticals (if any) were available for purchase and engaged in informal conversations with both vendors (to ask about details of their business operations) and with customers, to explore their motivations for patronizing particular sellers. We spent up to 2 hours at each market and wrote detailed observational fieldnotes immediately after.

In addition, the main study [21] entailed face-to-face focus group discussions (FGDs, *n* = 73 with a total of *n* = 611 individuals) and key informant interviews (KIIs, with *n* = 111 informants) in urban and rural sites across each of the four countries. Separate FGDs were conducted with men and women (an equal number of each), and participants were recruited with the help of a local gatekeeper who ensured a diverse range of ages, backgrounds and experiences was represented. Key informants included individuals with knowledge of medicine availability and use within each community, including community leaders and local healthcare professionals. The FGDs and KIIs were structured by first discussing experiences of medicines within the community, including where people obtained medicines, why there, and any concerns they had about the quality of their medicines (Supplementary File). No new themes or insights emerged in the latter stages of data collection, indicating a comprehensive coverage. Interviews and FGDs, which lasted approximately 30–60 minutes, took place in quiet public spaces and were conducted in relevant local languages and were audio recorded with the participants’ permission. In this paper, we report on the subset of data in which participants discussed the informal medicine sellers within their communities and the reasons underpinning their purchasing decisions. A summary of the research activities in each study site (including numbers of FGD participants and key informants) are shown in Table 1.

### Analysis

Fieldnotes from the informal markets were typed up and FGDs and key informant interviews were transcribed from the audio recordings and translated into English where necessary. Thematic analysis [22] was undertaken in NVivo, led by one researcher [JW] in consultation with coauthors, using an inductive approach with themes and insights derived from the data rather than being imposed. The process involved 1) familiarization through repeated reading of transcripts and fieldnotes, 2) identifying initial codes, with particular attention to excerpts on informal medicine providers, 3) collating codes into potential themes, 4) iteratively refining the themes against the dataset, and 5) producing the findings with illustrative examples. These examples included key quotations to support each theme and case studies to further illustrate the themes’ interconnections and complexities. Coding of data on reasons for patronage was conducted independently by two authors (JW and KH), with discussion at regular intervals to ensure consistency and reliability.

### Ethical considerations

Ethical approval was granted by the Research Ethics Committee in the Department of Anthropology at Durham University (reference: *ANTH-2022–06-06T10_50_51-pzft66*) and from in-country review boards in Ghana (University of Cape Coast IRB, reference: *UCC/IRB/A/2016/1594*), Nigeria (National Health Research Ethics Committee, reference: *SS1825ES*), Sierra Leone (Njala University IRB) and Uganda (Mildmay Uganda Research Ethics Committee and the Uganda National Council for Science and Technology, reference: *NHREC/01/01/2007–18/05/2023*). No personally identifiable data were collected in this project. Participants gave verbal consent prior to participation; written consent was not appropriate in these contexts where literacy rates are low and there is often suspicion of ‘official’ forms. This aligns with the current Association of Social Anthropologists of the UK (ASA) ethical guidelines which emphasize the importance of seeking consent in a way that is appropriate and meaningful to participants. All research was performed in accordance with the Declaration of Helsinki.

## Results

In what follows, we 1) describe the informal medicine sellers, 2) discuss reasons for patronage as identified in the thematic analysis (making medicines affordable, convenience and accessibility, and social relationships], and 3) bring these themes into conversation with each other via case studies to illustrate the ways in which participants actively negotiated these reasons in the contexts of their circumstances.

### Description of the informal sellers

Throughout fieldwork, we observed (from participant observation) or heard descriptions (in the FGDs and key informant interviews) of four broad categories of informal sellers: weekly market stall-holders, itinerant peddlers, roadside vendors, and those selling medicines within general provision shops.

#### Sellers at weekly markets

Of the eight markets we visited, we found pharmaceuticals for sale in all but two (both in Uganda; these are not described further here). The markets in Ghana and Nigeria were fully outdoor, while the markets in Sierra Leone were indoor-outdoor hybrids. The markets ranged in size, from tiny with just a few rows of stalls, to sprawling with rows upon rows of sellers. A variety of items were being sold at each of the markets, including food, clothing, household items, toiletries, and medicines. In general, the selling and buying of medicines in the markets were done very openly with little attempt to conceal what was happening. They were not difficult to find and openly advertised their wares (via megaphones, for example, at one market in Sierra Leone).

**Figure 1.**
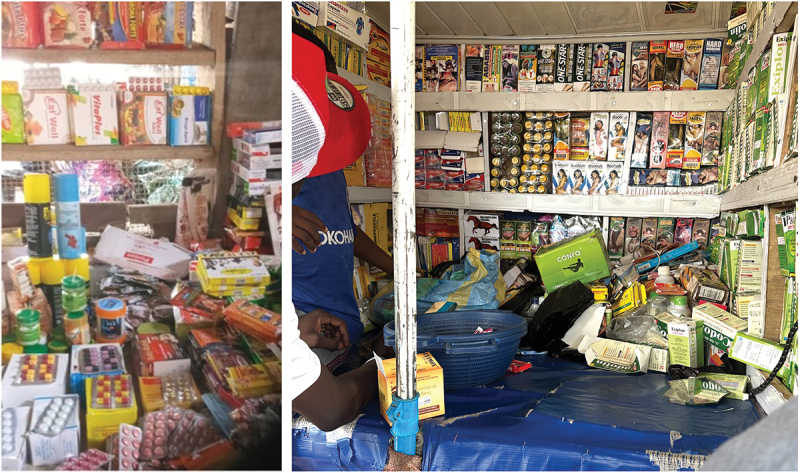
Market stalls selling pharmaceuticals in Sierra Leone [left] and Nigeria [right], taken with permission of the sellers.

The medicine vendors we encountered generally specialized in this, rather than selling other products alongside. In Ghana, the stalls were usually completely covered with an array of pharmaceutical and herbal medicines, most of which were labelled and marketed as products for sex-related issues. For example, we saw antibiotics (all indicated on the packaging as treatments for sexually transmitted infections), steroids (sold as body enhancers and appetite stimulants), herbal capsules and ointments for enlargement of the hips, breasts, and buttocks, and products similar to Viagra (sildenafil) for sexual performance enhancement. Most also had painkillers (including NSAIDs) available, but these were generally less conspicuously displayed than other medicines. In one case, for example, one seller kept the painkillers at the back corner of the table in a plastic basket that she rummaged through when a customer asked for this product; at another, we spotted painkillers in a bag on the ground behind the stall. In Nigeria, the situation was quite similar: herbal capsules and ointments for sexual performance enhancement predominantly lined the doors and walls of the cupboard of the stalls. Painkillers and antibiotics were also scattered across the tables, less prominently than the herbal medicines but still in plain sight (Figure 1).

In Sierra Leone, medicine vendors had a wide range of pharmaceutical products strewn across their tables (Figure 1). Products included paracetamol, antifungal creams, vitamins, as well as some herbal medicines. Antimalarials (including artemether-lumefantrine tablets and amodiaquine hydrochloride) were also widely available in one of the two markets. In some cases, these were concealed under tables because, according to the vendors, they could get arrested for selling them (although they were quite happy to show them to us when we asked). Others were displayed on stalls in plain sight.

#### Itinerant peddlers

Focus group participants and key informants in most (15 of 17) study sites spoke of itinerant peddlers regularly coming through their communities to sell pharmaceutical and/or herbal medicines, often door to door. Itinerant peddlers were particularly prevalent in urban and rural areas in Sierra Leone, where they were commonly referred to in Krio as ‘pepeh doctas’ (the term ‘pepeh’ (‘pepper’) apparently deriving from the pain felt on having an injection administered by these peddlers). We encountered two ‘*pepeh doctas’* roaming within an urban open market, one of whom was selling antimalarials (artemether-lumefantrine tablets) and paracetamol. According to FGD participants, *‘pepeh doctas’* were ubiquitous within their communities, regularly coming to sell paracetamol, *‘pain medicines’*, cold medicine, antimalarials, and antibiotics; others indicated some ‘*pepeh doctas’* sold only *‘not infections medicine* (sic), *but like for pain, headache, fever.’* One participant in Sierra Leone indicated some itinerant peddlers also sold medicines on public transport.

We did not see itinerant peddlers ourselves in the other three countries, but participants spoke frequently of them in Ghana and Nigeria. In Ghana, FGD participants in urban and rural sites described itinerant peddlers going house-to-house to sell medicines, sometimes on bicycles. Sometimes, participants referred to these sellers by their ethnicities, such as ‘*those Abokyi people’*, ‘*those Mossi people’*, and ‘*the Asantes*.’ Likewise, in Nigeria, participants spoke of hawkers (sometimes referred to as *‘abokis’*) roaming with medicines (particularly paracetamol) on their heads, in baskets, and in wheelbarrows in both urban and rural areas. In both countries, study participants also spoke of itinerant vendors operating on public transport; for example, ‘*sometimes you’ll see some people hawking or selling drugs from vehicles’* [Nigeria male, age unknown, urban Federal Capital Territory]; *‘When you are travelling to Accra, we have a lot of drug peddlers joining the car and selling their medicines in the car’* [Ghana 42-year-old male, urban Greater Accra] (see also [23]). By contrast, itinerant peddlers were spoken of relatively little in Uganda, although a few participants spoke of herbal medicines and sometimes painkillers being sold by ‘*people who move with cars’*, as well as peddlers on foot and on bicycles in mostly urban but also in rural areas.

#### Along the roadside

Several focus group participants in Nigeria, and a few in Ghana and Sierra Leone, also spoke of getting medicines by the roadside, particularly in urban areas. We visited two such roadside sellers in Nigeria. Both were located in an urban area in the Federal Capital Territory just outside the capital city (Abuja), in a large car park where taxis and commercial motorbikes and tricycles were parked and sometimes being repaired. There were several stalls dotted throughout the car park, most selling food and drink items and two selling pharmaceuticals. The first stand was a large cupboard, full from top to bottom, with neatly stacked boxes of paracetamol, antibiotics, and painkillers. The second stand, on the other side of the car park, was a small cart with medicines displayed across the surface. The medicines (all pharmaceuticals, mostly painkillers) were neatly stacked and most were in boxes, with a few cut blister packs in a basket. No customers came to either stall in the time we were there.

#### General provision shops

Several participants, mostly in Uganda, mentioned that provision shops often sell medicines informally, particularly paracetamol: ‘*even people who sell soap have paracetamol*’ [key informant, rural Mbarara, Uganda].

### Multiple and context-dependent reasons for patronage

Having outlined the various kinds of informal medicine vendors within the four countries, this section describes the diversity of reasons that people had for patronizing these sellers, drawing predominantly on the accounts of focus group participants plus our observations and informal conversations around market transactions.

#### Making medicines affordable

A key reason for patronizing informal medicine sellers was perceived affordability. Interestingly, medicines in the informal markets were usually no cheaper per unit compared with prices in private-sector pharmacies or licensed over-the-counter retailers. For example, in Sierra Leone, a packet of 24 tablets of antimalarials (labelled as containing Artemether 20 mg and Lumefantrine 120 mg, manufactured in China) was priced at Le20,000 in the market; the same product and dosage (under a different brand name and manufactured in India) cost Le18,000 in the pharmacy around the corner. Some informal vendors were reported to use dynamic pricing strategies to increase their profit when demand was high. For example, a participant in Sierra Leone said *‘the pepeh doctas can change prices, for example by dropping the price to draw business when they are new, and then increasing the price after that.’* [<25-year-old woman, urban Western Area Rural]. Others, especially those operating in rural areas, reportedly took advantage of high demand and lack of alternatives to keep prices high:

The demand is so high. If I go to the hospital [in the next town 16 miles away], I am able to buy paracetamol there for Le2000. The pepeh doctas sell it for Le5000, Le4000, Le3000. The profit is so high. [Male, age unknown, rural Bo, Sierra Leone]

xHowever, although the per-unit costs of medicines from informal sellers were not necessarily cheaper compared with the formal sector, customers were able to bypass other expenses associated with the formal sector, such as consultation fees and diagnostic laboratory tests as well as transportation costs, which were prohibitively expensive for many:

Sometimes such a person doesn’t have money or even health insurance to go to see a doctor, so with the little amount that the person has, he/she would want to manage and use it to buy from the roadside or drug store so that some can still be left to feed on. [Female, age unknown, rural Upper East, Ghana]

Moreover, informal vendors typically offered customers flexible modes of dispensing and payment that increased affordability. First, informal sellers of all kinds would routinely dispense the quantity of medicine according to how much participants could afford at a given time, irrespective of dosage requirements:

What they consider there [when buying from hawkers] is the money. They can just tell them to give them one dose. They will cut it [the blister-pack] for them. It’s affordable. [Female, urban Federal Capital Territory, Nigeria]

Second, many informal sellers, particularly those in fixed posts such as markets but also some itinerant peddlers who repeatedly came to specific communities, would allow familiar customers to buy medicines on credit. A market seller in Ghana explained to us:

Many people will not have money. Over-the-counter shops don’t sell on credit, but we can; if we know the person, they can bring the money on the next market day. [Author fieldnotes, 1 December 2022]

Finally, informal sellers could negotiate prices with individual customers, allowing additional flexibility compared with pharmacies. We experienced this ourselves: a stall-holder in Sierra Leone offered us a discount of Le10,000 if we bought two packets of antimalarials, originally priced at Le20,000 each.

The relative affordability of medicines, associated with flexible dispensing and payment modalities, played a central role in participants’ patronage of informal sellers. Most were aware that these medicines might not be quality-assured, with implications for their potential effectiveness, but severe financial constraints reportedly left them with no other choice:

When they [pepeh doctas] come it is easy to afford, very cheap, so we are going for them. They are against our own goals, but we are not able to control them … More or less we are conscious that some drugs – they are expired, they are counterfeit drugs, but if you don’t have any other means, you are going for them. [28-year-old male, urban Port Loko, Sierra Leone]

#### Convenience and accessibility

Relative convenience of informal medicine sellers was cited by participants as another key reason for patronage. In some rural areas, with no licensed retailers and frequent public-sector stockouts, itinerant peddlers became the only option. For example, in one study site in rural Sierra Leone, the only nearby public-sector health facility regularly experienced medicine stock-outs, and the closest licensed medicine retailer was in a town 16 miles away. Thus, the itinerant peddlers who regularly passed through the community offered a way to avoid the substantial time and financial costs of travelling further afield:

Every day ‘pepeh doctas’ will come and then we buy medicine from them because we don’t have a good pharmacy here, no pharmacy, no good health center, and then the cost of the transportation [to the nearest town] is very high. [30-year-old male, rural Bo, Sierra Leone]

Medicine accessibility becomes even more pressing in acute or urgent cases, especially in rural and remote areas, where the choice could be between sourcing medicines from itinerant peddlers or doing nothing at all:

In the villages, people don’t have any options. When you are sick, it is a matter of desperation. So people will still buy these fake medicines [from peddlers] in their desperate attempts to get healed. [43-year-old male, urban Greater Accra, Ghana]

Convenience was also a factor in urban areas, but in rather different ways. Although the distances to formal-sector facilities were typically much shorter in towns, itinerant peddlers nonetheless offered added convenience of coming into communities, sometimes even directly house-to-house, sparing participants a journey:

For them to stand up and go to the chemist is very far. To go to the hospital is very far. When somebody is feeling headache and someone selling is passing by, they just go and buy from them. [Key informant, urban Abuja, Nigeria]

Study participants also noted the convenience of purchasing medicines from centrally-located markets or grocery shops while going about everyday business, in contrast to making a special visit to a health centre, with long wait times:

If I go to the health center, I will spend two hours there. If a person just wanted to cure headache, they will just go buy paracetamol in a [general] shop, and then continue to town and do their business. [33-year-old male, urban Mbarara, Uganda]

Indeed, market vendors benefitted from the convenience factor, which enabled them to pick up passing trade, as this female stall-holder in Ghana recounted:

Many of my customers are passing trade. They didn’t go to the market specifically to buy medicines, and so would not have gone to an over-the-counter or pharmacy shop. Instead, they are just in the market and see the medicines on my stall and decide to try one. [Author fieldnotes 3 December 2022]

#### Social relationships

Finally, both informal vendors and their customers spoke often about the social relationships that underpinned their transactions. Trust and familiarity played a key role in this (see [24,25]), especially for vendors who had been operating for a long time and are natives of the communities. This applied particularly to those working from fixed locations, such as a grocery shop or a regular weekly market stall. For example, one regular market-stall holder in Ghana attributed her success in part to *‘[the community] know[ing] me – I’ve been selling here for a long time so they trust my medicines are good’* [author fieldnotes, 3 December 2022].

By contrast, itinerant peddlers tended to be viewed more skeptically, because their mobile nature meant they could not be traced in the event of something going wrong:

This is one of the problems of buying medicines from people who come to the community to sell medicine: if anything happens, you cannot trace the people. [35-year-old female, rural Greater Accra, Ghana]

This uncertainty was exacerbated when the community had high levels of illiteracy, because it meant buyers could not necessarily verify what was being sold to them:

Sometimes I get worried about [itinerant peddlers], because I don’t know the people. I don’t know where they are coming from and what their intentions are concerning the medicines. It can be poison they give to people; you will not know, and because our people cannot read and write, they can buy anything ignorantly and that could worsen or complicate their health conditions. [40-year-old female, rural Upper East, Ghana]

However, itinerant vendors who were either natives of the community, or who had regularly revisited the same communities over an extended period of time were able to build up relations of trust: ‘*Most people buy from the same ones regularly’* [Key informant, rural Western Area Rural, Sierra Leone]. Indeed, one key informant (health worker) noted that the familiarity with certain itinerant peddlers made it difficult to persuade people to stop buying from them:

The drug peddlers are natives of the community, so it becomes very difficult to convince some of the community members to stop patronizing their services. [Key informant, rural Port Loko, Sierra Leone]

Second, some participants noted that their interactions with informal sellers were often more empathetic than those with health professionals. In contrast to their experiences in busy health centers, where they sometimes felt rushed or not taken seriously, informal sellers made them feel important and cared for, attending to them promptly and taking time to listen to them and to explain things carefully:

I go to the hospital but the health workers do not pay attention to me when I visit the hospital. But the ‘pepeh doctas’ are very prompt with their treatment and they are also attentive to our story and health. [20-year-old female, urban Port Loko, Sierra Leone]

Relatedly, some participants felt a greater sense of confidentiality with informal vendors compared with a perceived lack of privacy in the confined spaces of a busy hospital:

We go to [pepeh doctas] because we have our privacy but when you visit the hospital you do not have that privacy because people are always around us. The pepeh doctas are very confidential, unlike the doctors at the hospital who may not be trusted with some confidential health information. [28-year-old female, urban Port Loko, Sierra Leone]

Finally, shared cultural backgrounds could reportedly encourage patronage, especially where buyer and seller shared a common language or ethnic identity:

You will see these Hausa people—mostly they patronize [hawkers] because … they speak the same language. [Female, rural Federal Capital Territory, Nigeria]

### Tradeoffs and multiple logics

As we have shown, financial flexibility, convenience, and social relationships were strong influences on patronage of informal medicine sellers. However, it is too simplistic to assume that all three always applied. In reality, the relative importance of these factors, and the ways they operated, shifted according to circumstances in a given place and time, with patrons having to negotiate the priorities in each specific situation. The following three case studies demonstrate the ways in which the logics for patronage were considered and the tradeoffs that underpinned purchasing practices. All names are pseudonyms.

#### Case study 1: John^1^ in rural Sierra Leone

John is a 32-year-old electrician living in a small, rural town in the Bo district of Sierra Leone. This community of about 10,000 people is approximately 15 miles from the nearest urban centre, the district capital. However, the collapse of a bridge 6 months prior to our fieldwork means that the journey to the district capital now requires hiring a boat to cross a narrow river, and then securing a motorbike taxi at the other side: an arduous, expensive and time-consuming endeavour.

John’s town is big enough to have a government health centre, but John and others tell us there are very rarely medicines in stock there. There are no licensed retailers operating in the town, but there are itinerant ‘pepeh doctas’ who come on a regular basis. John is wary of buying medicines from the peddlers because he is not confident in their knowledge about the medicines or their efficacy: *‘They don’t know about medicine. If someone is feeling headache, they don’t know what type of medicine they are supposed to give to the individual … but sometimes the medicine can work.’* He also explains that peddlers’ prices are higher than the pharmacy, and they do not sell on credit; they will, however, cut and sell the number of tablets he can afford, rather than insisting on dispensing a full dose: *‘If the pepeh docta tells me that this medicine is Le10,000 and I have only Le5000, he can separate it and put [what I can afford] in the plastic.’* John buys medicine from these ‘pepeh doctas’ on a regular basis. For him, it is a matter of proximity; even though the prices are higher and he does not fully trust their knowledge or medicines, this is his only option to spare a long and (for him) unaffordable journey to the city.

#### Case study 2: Emmanuel in Nigeria

Emmanuel is a 45-year-old carpenter living in a densely populated urban settlement adjacent to Nigeria’s capital city, Abuja. Within the community, there is a general hospital, a primary health care centre, a private hospital, and pharmacies or licensed retail outlets on nearly every street. There are also many informal medicine sellers, with mobile stalls set up in car parks and along the roadside. Emmanuel expresses concerns about the medicines they sell, noting that

you don’t know if it’s expired because there are some drugs that they will remove the evidence of the date of manufacturing and expiring. Some of them do that … you can’t ascertain the quality. But I buy them sometimes.

When asked why he purchased them despite his concerns about their quality, Emmanuel replied, ‘*because their drugs are cheaper … To go to hospital now, it will cost you no less than NGN5000. If you have NGN500, you can still manage medicine from these roadside people.’* He also noted that, unlike the pharmacy, roadside sellers would sell partial doses according to what he could afford:

Assuming I have a case in which I will need an antibiotic, the duration will be between five days to maybe ten days. Now the pharmacy store will insist on me buying the right dosage for the right duration. But for these hawkers, they can decide to just cut one tablet … Why would I go to the pharmacy store that I have to pay NGN1000+, meanwhile these hawkers will just cut the tablet for me at NGN300 or NGN200?

For Emmanuel, informal sellers solve the issue of affordability; despite his concerns about the quality of their medicines, and the proximity of formal-sector outlets, the prohibitive user fees and the costs of full courses of medication make informal sellers a local, affordable option.

#### Case study 3: Esther in Ghana

Esther is a 49-year-old hairdresser living in an urban community in the Upper East Region of Ghana. There is a hospital nearby and a licensed medicine retailer within a ten-minute walk from her home. Esther tells us she usually sources medicines from the licensed retailer because, when she attends the hospital, they are usually out of stock of medicines. Itinerant peddlers sometimes come through her community but she says *‘it is very risky to buy medicines from them.’* There is a large market nearby that operates every 3 days, with traders coming to sell food, clothes, household goods, and medicines. When Esther visits the market to purchase food, she sometimes passes by a medicine seller named Sophia, who has been operating at this market for 20 years. Esther, like many community members, has got to know Sophia over the years and has grown to trust her. Sometimes, as Esther passes through the market, she will stop by Sophia’s stall to purchase painkillers, so she has some on hand the next time she or someone in her household has a headache; other times, she will purchase medicines for others at their request. On days where Esther does not have sufficient money, Sophia is willing to sell to her on credit because she knows her and trusts she will repay when she can. For Esther, patronizing informal medicine sellers is primarily about the trust she has in one particular seller (not the informal sector as a whole), with the added convenience and flexibility to purchase on credit if need be.

## Discussion

This study aimed to describe the informal medicine sector in four anglophone African countries and understand the reasons that people patronize unlicensed sellers. The key findings were that both the informal medicine sector, and peoples’ reasons for patronage, are multiple and contingent. The informal medicine market is not a single phenomenon; it is segmented and diverse, with multiple actors and products serving a range of niches. Reasons for patronage are similarly complex and contingent, as the urgent need to source affordable medicine becomes enmeshed with social relations of trust and care.

Many of the reasons participants in this study had for patronizing informal markets have been described elsewhere, and relate principally to limitations in formal health systems (flexibility, availability, accessibility). For example, the comparative affordability of the informal market, particularly through mechanisms of being able to purchase medicines on credit and/or only in smaller quantities, has been previously noted in several African countries, as have the roles of convenience and accessibility [4,12,18,19,26–28]. A novel insight identified in this study is the tradeoffs and tensions among these motivations and drivers. As the case studies illustrated, patrons actively weighed these tradeoffs and made decisions about where to obtain medicines on the basis of what was accessible or important to them. Most expressed reluctance to source medicines from the informal sector but had little choice because the formal sector was out of their reach for reasons such as unaffordability or inaccessibility. For others, patronage of specific actors within the informal sector was driven by trust in particular vendors, stemming from longstanding relationships or shared cultural backgrounds [24,25].

This nuanced and contextualized understanding of the informal medicine market has several implications for policy and practice. Efforts to tackle the proliferation of informal medicine markets in LMICs typically centre on a combination of tighter regulation and public education about the dangers of frequenting unlicensed vendors. While these are both important, their effects are likely to be limited without addressing the root causes of the underlying demand. This is particularly the case in resource-constrained contexts, where regulatory capacity is limited, and where the demand for products from unlicensed vendors is driven more by necessity than by ignorance of the risks. While people lack other (realistic) options, the demand for informal medicine vendors is likely to remain high, regardless of public education campaigns or similar interventions. Indeed, more stringent regulation of informal sellers without concurrent efforts to increase access to products from official sources may end up cutting off the only source of medicines for some segments of the population, particularly the poorest and most rural [29], thereby potentially exacerbating healthcare inequities. Similarly, public education campaigns that fail to appreciate the constraints that drive people to source medicines informally may backfire unless realistic alternatives are available [21].

Reducing the demand for medicines from unlicensed vendors therefore necessitates first and foremost expanding effective access in the formal sector. The findings in this paper highlight a range of ways in which the informal sector effectively meets the population’s needs, offering potential lessons for the formal sector. Crucially, there are always medicines available in the informal sector. Ensuring adequate supplies of medicines to public-sector health facilities, especially in rural areas, is an absolute prerequisite for reducing the demand for informal sellers. The informal market is also extremely adept at rapidly responding to the needs of the specific context and population it serves. For example, itinerant peddlers travel to communities who lack alternative sources of medicines and spare customers from making special journeys by coming to their houses or other convenient locations. Some market sellers respond to patrons’ inability to pay upfront by offering to sell on credit, and informal sellers tend to make customers feel heard and important.

Drawing on these lessons, access to medicines within the formal sector may be enhanced by (for example) offering flexible modes of payment, investing in more effective delivery mechanisms to underserved areas, increasing convenience (bearing in mind other demands on people’s time), and making patients feel prioritized and valued. In practical terms, this might entail making greater use of trained community health-workers (CHWs) to prescribe and dispense basic medicines as part of their core activities, capitalizing on their familiarity and acceptance within the communities they serve. This fits broadly within the ‘task shifting’ umbrella, whereby tasks are redistributed to health-workers with lower levels of qualification, as a way of increasing provision, especially in low-resource settings [30,31]. However, the capacity for CHWs to take on additional responsibilities indefinitely is not unlimited [2,32]; any such initiatives would need to be properly resourced and accounted for in CHWs’ workloads.

A second (complementary) set of approaches would be to acknowledge and leverage the presence of informal medicine markets in these contexts, by integrating them into initiatives to improve medicine access, rather than seeking to eradicate them [33,34]. This could range from providing basic training to informal/itinerant vendors, to ensure they have at least a minimum understanding of the products they are selling, to more ambitious schemes that entail certification and licensing of individuals who engage in ongoing training and compliance with basic standards. This could provide a potentially effective compromise for those who (for whatever reason) rely on informal sellers to supply their medicines, but who would want some reassurance about their level of expertise and quality of their products. However, any such initiatives would need careful planning to avoid the risk of adding yet another layer of complexity and ambiguity in an already-murky pharmaceutical retail landscape.

The findings of this study should be considered in light of its strengths and limitations. This study used multiple qualitative methods to provide a nuanced description of the informal medicine sector as well as context-specific reasons for patronage in four different countries. While participant observation of markets and roadside sellers occurred only in urban areas, focus groups and interviews were conducted in both urban and rural locations, capturing views and experiences beyond urban centers. The descriptions of the informal sector here are not necessarily exhaustive; however, as this work was conducted as part of a broader study on substandard and falsified medicines. Additionally, as there was an uneven balance in evidence between countries, any comparisons should be made with caution.

## Conclusion

This description of the informal medicine sector in four anglophone African countries has highlighted that the informal market is segmented and complex, and people have multiple logics for patronizing it. Affordability, relative convenience and accessibility were key reasons for patronage, often embedded in social relationships of trust. However, tradeoffs and tensions between these reasons were common, requiring people to negotiate priorities within their current circumstances. The underlying drivers of informal medicine markets derive from limitations of the formal medicine sector, which mean that some sectors of the population (typically the poorest and most rural) must make invidious choices between medicines of uncertain quality/dosage or no medicines at all. An equity-informed approach must therefore prioritise improving affordability and accessibility of quality-assured medicines in the formal sector. Without such investment, any attempts to control informal-sector sellers are likely either to fail or to further reduce access to essential medicines for the most vulnerable.

## Supplementary Material

COREQ_Checklist.pdf

Supplementary File.docx

## Data Availability

The data underlying this article will be shared on reasonable request to the corresponding author.
