# Supplementary material for: Logics of acquiring medicines from informal retailers in four African countries
Source: Glob Health Action. 2025 Oct 29;18(1):2574764. doi: 10.1080/16549716.2025.2574764 (PMC12573556; doi:10.1080/16549716.2025.2574764)
Supplement: Supplementary File.docx [file ZGHA_A_2574764_SM3075.docx]

# Indicative Focus Group Guide for use in Community Settings

1. **Project introduction (summarising the project, house-keeping, confidentiality) and seeking consent**
2. **Participant introductions and collection of background information** (e.g., gender, occupational status)
3. **Where do people get medicines and why there?**
   - Where do they usually get any medicines they or their household members may need? Why do they go where they go?
   - Do they ever experience difficulties getting the medicines they or their family need? If so, what kinds of difficulties?
   - How confident are they that the medicines they get are of good quality? How do they know?
4. **What do participants know about SF medical products and how?**
   - What have they heard from different sources?
   - Do they have any direct or indirect experience of potentially SF products?
   - Do they feel it’s something that affects them? Is it important to them? Is it a source of concern?
5. **Have participants seen any of the risk communications from the WHO-led project?** [Ask each individual to respond and tabulate responses]

***[Show the materials to all participants]***

1. **Discuss reactions to the materials and messages**
   - What are their reactions to the campaign materials?
   - What do they think the materials are trying to say?
2. **Discuss potential impacts of the messages on medicine-related behaviours**
   - If this message was shown more broadly, what do they think other people would do in response? Would other people understand it? Would it change anything about what people do?
   - What things might make it easier for people to respond to these messages? What things might make it harder?
3. **Discuss the possibility of (negative) unforeseen consequences of the campaigns**
   - Might people change their behaviour in a worse way rather than a better way?
   - Are there any other possible unforeseen consequences? [Begin with open-ended question but be prepared to prompt about risks of undermining trust in medicines / healthcare providers more generally]
   - Overall, what do you think the impact of showing these materials might be?
4. **Ask about awareness of previous medicine incidents/recalls**

- Have people ever heard of cases/incidents where a medicine has been recalled?
- [If a prompt is needed, did people hear about the cough syrup incidents in the Gambia last year and Cameroon this year in which children died because the cough syrups were contaminated?]
- How did people hear about such incidents?
- How did they react to the news? Did it cause any changes in their thoughts or actions related to medicines?

1. **Brainstorm next steps**
   - Going forward, what do you think the most sensible thing to do is to help ensure that the medicines people get are good quality?
   - [If people mention communication campaigns as potentially useful or important], how should the messages be presented to be most meaningful and accessible?
2. **Closing discussion**
   - Follow up on any questions, threads and leads to get more depth as required
   - Ask for any additional comments, thoughts and reflections
   - Thank the participants and close
